# Progress in Anæsthetics

**Published:** 1901-07-06

**Authors:** 


### PROGRESS IN ANÆSTHETICS.

THE report of the Anæsthetics Committee of the British Medical Association[1] contains much of interest, though that more dogmatic teaching is not forthcoming must be disappointing to many. Several statements are striking: in the first place, chloroform is credited with being the most dangerous anæsthetic in use, however employed, in mixtures or otherwise. Evidence tends to show that special danger is incurred when it is administered on a closed inhaler (Clover's). And the administration of it on a towel seems more fraught with danger than on lint. Chloroform is about twice as dangerous in males as in females, and it is most dangerous in infancy and after thirty years of age. In males percentage of danger cases under chloroform was nearly six times that of danger cases under ether, more than seven times that of danger cases under gas and ether, and nearly two and a half times that of danger cases under the A.C.E. mixture. In the female cases the percentage of danger cases under chloroform was nearly twice the danger cases under ether, more than twice that of danger cases under gas and ether, and nearly twice that of cases under the A.C.E. mixture. With patients in good condition one "danger case" occurred in every 133 administrations of chloroform, in every 654 of ether, and in every 834 of gas and ether.

Danger to life is especially liable to occur at the early periods in the administration of anæsthetics. There is evidence to show that the danger under chloroform is due to primary cardiac failure, while imperfect anæsthesia is responsible for a large number of cases of danger under chloroform. Anæsthetics are notably more dangerous in proportion as the gravity of the patient's state increases. The material before the committee did not justify them in making more definite statements; the working of anæsthetics was a large personal factor, not only in the patients', but in the administrator's judgment and experience. It is not surprising, therefore, to see that the committee lay great stress upon the experience of the administrator, and urge the more routine teaching of anæsthetics to all students, and the choice, where possible, of those whose special attention has been devoted to the subject, in all difficult cases.

Dr. Waller's[2] criticism upon this report is valuable in that he points out that the calculation of the danger rate of ether, from three inadmissible cases, is misleading, and therefore the comparison of this anæsthetic with chloroform in the number of cases forthcoming is not trustworthy. He also compares from the report the relative danger in the methods of administering chloroform, giving, in order :—

| | Total number of cases. | Danger or deaths. | Rate per minute. |
|---|---|---|---|
| Junker .. .. .. .. | 411 | 0 | 5·58 |
| Open method .. .. .. | 326 | 0 | not given |
| Lint.. .. .. .. .. | 3,793 | 23 | 8·25 |
| Skinner's mask .. .. .. | 2.375 | 25 | 8·86 |
| Towel .. .. .. .. | 4,441 | 65 | 10·6 |

This table seems to show that more danger exists the more rapid the administration, and, therefore, the higher the percentage of vapour.

In the *Lancet* report[3] on anæsthetics there is evidence that alcoholism is one of the dangerous and frequent predisposing causes of accidents under chloroform, while the same condition was the cause of complications after ether administration, though not adding to the immediate danger. Emphysema is next dangerous, while a high danger rate is associated with shock, collapse, affections of the heart, empyema, bronchitis, and anæmia. Vomiting during anæsthesia is a cause of considerable danger, and it would seem also a cause of complications. There are several points of divergence from the British Medical Association Committee's report, though both reports agree in placing operations on the tongue and upper air passages as peculiarly dangerous, while mishaps in the operations for strangulated hernia seem to be more due to the stercoraceous vomit than to any particular anæsthetic used. Another conclusion is that when mixtures containing chloroform, *e.g.*, the A.C.E. mixture, are exhibited from closed inhalers the danger rate is at once greatly increased. The *Lancet* report evidences the variety of fatal lung complications after ether inhalation. When given in cases of bronchitis or emphysema ill results have occurred. Renal symptoms are conspicuously absent as a direct result of ether inhalation. Circulatory failure is of far more dangerous import than respiratory in chloroform narcosis.

That death is due to failure of the heart in chloroform narcosis, is proved by Professor Simon Duplay[4] and Dr. Louis Hallion, who in their experiments on animals, have shown that when the respiration has failed, if artificial respiration is substituted, while the anæsthetic is continued, the circulation comes to a stop in spite of it. Thus, arrest of respiration although not serious in itself, is nevertheless a very important indication of the nearness of the border line, over which the circulation is depressed beyond control. They also have made experiments showing that the blood pressure under ether narcosis remains normal or even above normal, but in chloroform narcosis the initial rise gives place to a considerable, and possibly dangerous, fall.

Hewitt[5] shows that by enlarging Clover's narrow air ways, using an apparatus with a wide bore, stertor and cyanosis are largely eliminated from the administration of gas and ether. Stertor he holds to be explained by three theories:—1. That it is due to vascular turgescence, as when the tongue is engorged during asphyxia. 2. That it is spasmodic, from spasm of the muscles of the upper air tract. 3. That it is due to the paralytic condition of the soft parts allowing them to fall together and occlude the passages. It is possible for the spasmodic and vascular stertors to occur together, or for one to pass into the other, especially during asphyxia. The large-bore inhaler does not permit of asphyxia, which is so dangerous in some cases. He recommends the nitrous-oxide ether-chloroform sequence in anæsthesia, holding that the dangerous stage is passed under the safe ether, while the after results of chloroform are not so unpleasant or deleterious to the lungs, &c., as prolonged ether administration. But he warns against using the chloroform after deep anæsthesia with ether, or in the struggling stage. He allows the patient to return to the point of reflex when he will clear the throat of any mucus lodged there; this has the result of preventing any respiratory embarrassment later. In this way much chloroform is not required, except in abdominal cases.

Ethyl chloride, or "kelene," has now been used for over two years as an anæsthetic—2,550 cases having been collected by G. Lothrissen,[6] who gives as his opinion upon its use that it is very useful in short operations; as far as fatality is concerned, statistics show it comes between chloroform and ether, yet no specific injurious action of the drug, even after long narcoses, is observed, the kidneys not being affected. That lengthy administrations in cases of heart and lung diseases have had no injurious result, as would certainly have occurred under ether. In alcoholic cases he prefers to inject muriate of heroin to prevent undue excitement and insure complete muscular relaxation during its use. Vomiting does occur after prolonged narcoses, but never so violent as after chloroform or ether, and with no unpleasant after-results. The patients do not complain of unquenchable thirst, but rather of a feeling of hunger. He advises that the drug be only used in small doses (at the most 3 grs. at the commencement), and a mask with inspiratory and expiratory valves. During excitement the administration should be suspended until the expired air contains no more odour of ethyl chloride. He says: "Only if minute and exact attention be paid to the narcoses do I consider it to be without danger. If marked excitement occurs, or the face becomes cyanotic, the mask must be immediately removed, and the face rubbed with a wet, cold towel."

The literature on the subject of intra-arachnoid injections of cocaine for producing anæsthesisa, is summed up by Dumont,[7] who comes to the conclusion that it is at present unjustifiable. Bier, who introduced it, injected through a needle, introduced as in lumbar puncture, about $\frac{1}{13}$ to $\frac{1}{6}$ grain into the subarachnoid space. On cerebro-spinal fluid returning through the needle, the cocaine was injected. Operations are quite painless on the lower part of the body and limbs, although tactile sensation and knee-jerks are retained. The untoward results comprise nausea and vomiting, tachycardia and violent headache, while Dumont, Saldowitsch, and Zaider record high temperature, 104° Fahr., rigors and insomnia. It seems to be not wholly free from danger to life, as a case of Kocher's died with symptoms of meningitis. Tuffier, however, holds that it is generally applicable; he has used it for nephrotomies, nephrectomies, vaginal hysterectomies, herniotomies, and removal of the vermiform appendix, in all in sixty-three operations without a death or other mishap than some of the symptoms recorded above, such as transitory high temperature and rigors. Lewin[8] distinguishes between the vomiting due to the contact of chloroform with the stomach, brought about by swallowing saliva impregnated with it, and the later vomiting due to elimination of the chloroform dissolved into the circulatory system. He recommends dilute cocaine to be administered before an anæsthetic, and mechanical protection of the stomach by means of mucilaginous material.

In order to get rid of the nauseous after-taste of ether, Silk[9] recommends removing the gastric and buccal mucous and saliva as soon as they accumulate by wiping out the mouth and putting a strip of gauze or wool into the dependent angle of the mouth. After return to consciousness let the patient wash out his mouth and drink copiously of hot water. The excessive dread of anæsthetics shown by some patients adds to the difficulty and danger of the administration. Feilchenfeld[10] finds that the exhibition of tincture of strophanthus for the previous two evenings and the same morning has a favourable result.

Loss of irritability of tissues after death is a varying quantity, and it has been found that the heart muscle will retain its irritability for some time after respiration has ceased and the pulse stopped. Prus[11] has experimented upon dogs, and by massage to the exposed heart has restored the beat and respiration for a few hours. He applied this also to a suicide, with the result that the heart was induced to beat two hours after death. In a patient who had died from chloroform, after all other means had been tried in vain, including forcing air into the lungs, the exposed heart was massaged and air driven in rhythmatically, with the result that respiration was restored 3½ hours after the operation was begun. For half an hour the respirations were conducted normally, and the radial pulse strong and regular. After the wounds were sutured, the respiration lasted for twenty minutes; then it became embarrassed and stopped. Air inhalations and other restoratives were used to no purpose. The heart continued to beat for eight hours. Kyer Peterson suggests a tampon catheter instead of the rough bellows they used, and so prevent air entering the œsophagus, and producing meteorism.

[1] Brit. Med. Jour., Jan. 19, 1901; Brit. Med. Jour., Feb. 23 1901; Lancet, Jan. 26. [2] Brit. Med. Jour., Feb. 23, 1901. [3] Lancet, March 30, 1901. [4] Lancet, Oct. 20. [5] Lancet, March 30. [6] Birmingham Med. Rev., Dec. [7] Brit. Med. Jour., May 5. [8] Lancet, March 9, 1901. [9] Olin. Jour., Nov. 14. [10] Brit. Med. Jour., June 9, 1900. [11] Brit. Med. Jour., Jan. 19.

